# Nontuberculous Mycobacterial Infection Predisposing to Chronic Cavitary Pulmonary Aspergillosis

**DOI:** 10.7759/cureus.16418

**Published:** 2021-07-16

**Authors:** Bryan Vera Nieves, Geoffrey Lindblad, Benjamin Carmel, Andres Rivero, Simon M Edelstein

**Affiliations:** 1 Infectious Disease, Aventura Hospital and Medical Center, Aventura, USA; 2 Radiology, Aventura Hospital and Medical Center, Aventura, USA; 3 Internal Medicine, Aventura Hospital and Medical Center, Aventura, USA; 4 Infectious Disease, Aventura Hospital and Medical Center, Miami, USA

**Keywords:** chest tube, pigtail catheter, mediport, nontuberculous mycobacteria, aspergillosis, chronic cavitary pulmonary aspergillosis, atypical mycobacteria, mycobacterium avium intracellulare

## Abstract

*Aspergillus* is a large group of spore-forming fungi in the phylum Ascomycota. *Aspergillus* infections more frequently occur in individuals with pre-existing lung conditions such as cystic fibrosis and asthma and immunosuppressed individuals, and less frequently in the immunocompetent population. Pulmonary aspergillosis can be subdivided into three categories: allergic bronchopulmonary aspergillosis, chronic pulmonary aspergillosis, and invasive pulmonary aspergillosis. We present a rare case of a 57-year-old male with a previously known diagnosis of pancreatic adenocarcinoma on chemotherapy who was found to have a co-infection of the respiratory tract by *Aspergillus flavus* and *Mycobacterium avium intracellulare.*

## Introduction

Chronic pulmonary aspergillosis (CPA) comprises several different disease manifestations of chronic aspergillosis: simple aspergillomas, *Aspergillus* nodules, chronic cavitary pulmonary aspergillosis (CCPA), and chronic fibrosing pulmonary aspergillosis. CCPA, in particular, is characterized by one or more pulmonary cavities with or without aspergillomas, symptoms present for at least three months, and a positive *Aspergillus* immunoglobulin G (IgG) serology.

Mycobacterial infections are also capable of causing various pulmonary manifestations, particularly in immunosuppressed individuals. Both *Aspergillus* and *Mycobacterium* are opportunistic pathogens that can cause severe pulmonary disease, but co-infection of the respiratory tract with both pathogens is rarely reported in the literature [1]. It is estimated that up to 3 million people are affected by CPA worldwide [2]. Here, we present a rare case of a 57-year-old male with a previously known diagnosis of pancreatic adenocarcinoma on chemotherapy who was found to have co-infection of the respiratory tract by *Aspergillus flavus* and *Mycobacterium avium intracellulare*.

## Case presentation

**Figure 1:**
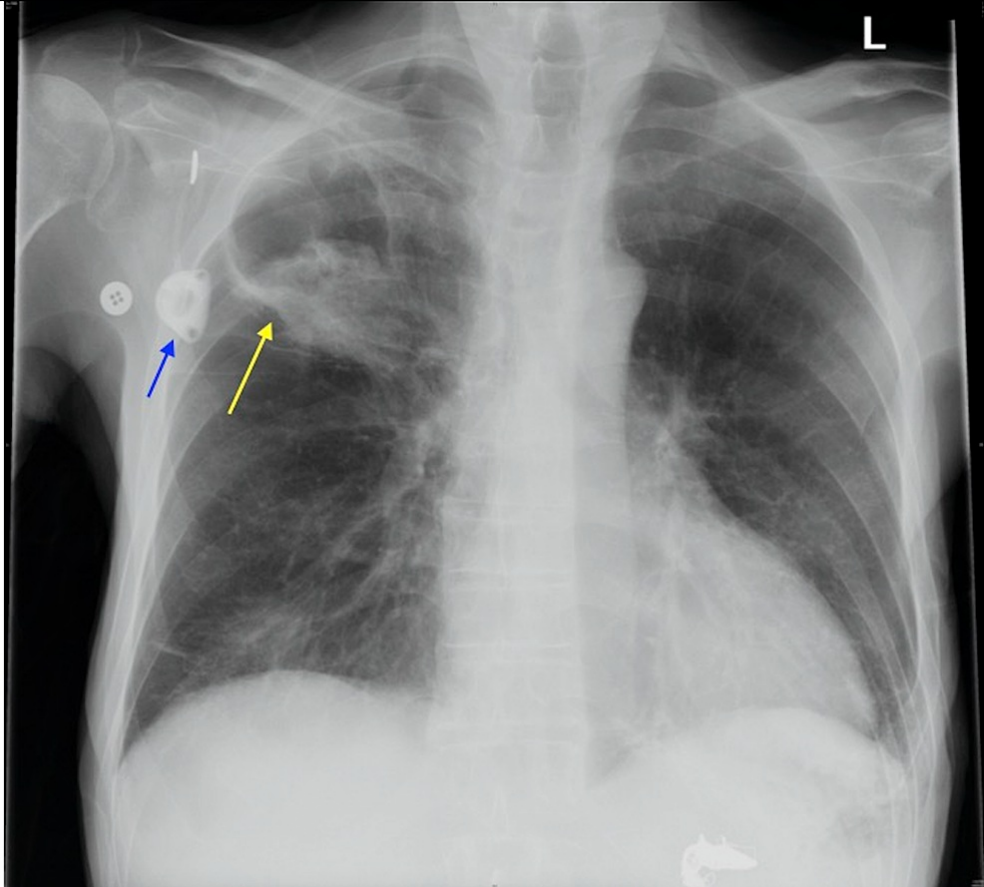
Chest radiograph demonstrating a right-sided apical cavitary lesion (yellow arrow) and a chemotherapy port (blue arrow).

A 57-year-old male with a past medical history pertinent for hyperlipidemia, pancreatic adenocarcinoma diagnosed six months prior and on chemotherapy, as well as recurrent bouts of pancreatitis presented to our institution due to shortness of breath, fever, chills, and epigastric abdominal pain radiating to his back and associated with nonbloody emesis that had been present for five days prior to arrival. Physical examination revealed a cachectic and frail man, with epigastric abdominal tenderness and voluntary guarding. Vital signs revealed a blood pressure of 102/63 mmHg, heart rate of 62 beats per minute, temperature of 36.9°C, oxygen saturation of 99% at room air, and a respiratory rate of 16 breaths per minute. Initial laboratory studies revealed an elevated lipase of 1671 U/L (10-140 U/L) and leukocytosis of 17.1 × 10^3^/uL (4.0-10.5 × 10^3^/uL) with a 75% neutrophilic predominance. A chest radiograph revealed a large right-sided apical cavitary lesion measuring up to 6 cm (Figure 1).

**Figure 2:**
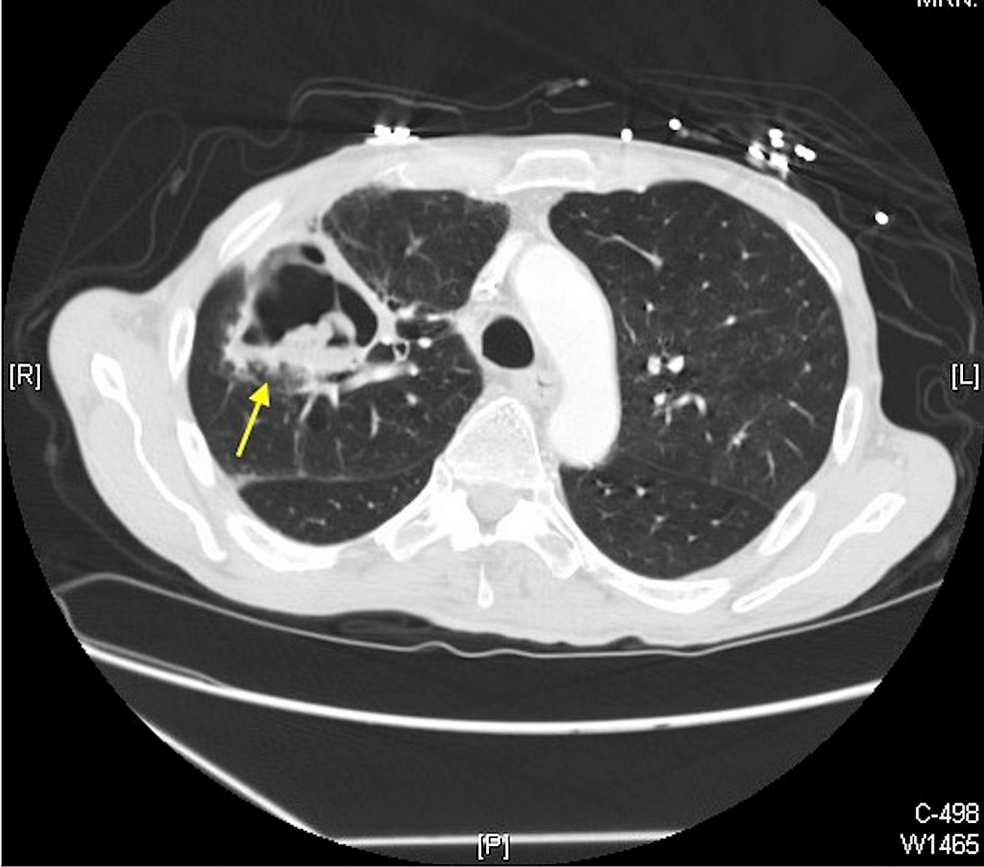
Axial reformatted CT chest with IV contrast demonstrating a cavitary lesion with internal dependent debris and an air-fluid level in the right upper lobe apical and anterior segment measuring 5.7 × 5.7 × 5.8 cm (yellow arrow). CT: computed tomography; IV: intravenous

A computed tomography (CT) scan of the chest with intravenous (IV) contrast was obtained to further characterize the findings evident on the chest radiograph which revealed a cavitary lesion with internal dependent debris and air-fluid level in the right upper lobe apical and anterior segment measuring 5.7 × 5.7 × 5.8 cm (Figure 2).

The patient was started on aggressive IV fluid hydration with Ringer’s Lactate for the treatment of pancreatitis as well as IV piperacillin-tazobactam to cover for the possibility of bacterial cavitary pneumonia.

Bronchoscopy with bronchoalveolar lavage (BAL) was performed and fungal culture grew *A. flavus *with fungal smear revealing septate hyphae branching at 45-degree angles. *A. flavus* titers were elevated at 1:4, for which a diagnosis of CCPA was made. HIV testing was negative. Antifungal therapy with voriconazole 200 mg PO BID was initiated. Piperacillin-tazobactam was discontinued as the BAL cultures were negative for bacteria. BAL acid-fast bacilli culture grew *M. avium intracellulare* and the patient was started on ethambutol 800 mg PO QD, azithromycin 500 mg PO BID, and amikacin 250 mg IV BID.

The patient clinically improved with decreased oxygen requirement and down-trending white blood cell counts and was discharged from the hospital on voriconazole, ethambutol, azithromycin, and IV amikacin.

**Figure 3:**
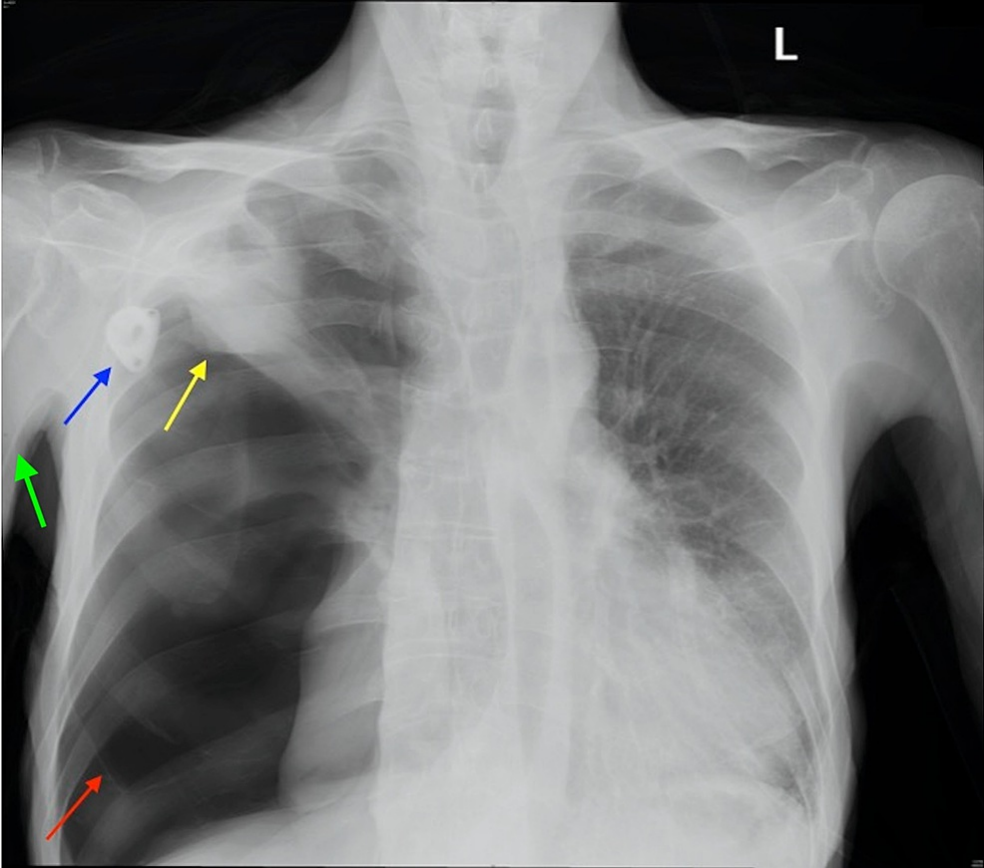
Chest radiograph demonstrating a right-sided pneumothorax (red arrow), a right-sided apical cavitary lesion (yellow arrow), a chemotherapy port (blue arrow), and a PICC line (green arrow). PICC: peripherally inserted central catheter

**Figure 4:**
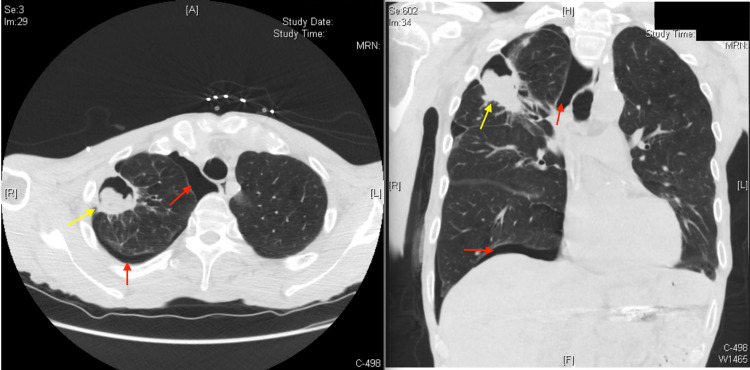
CT chest with IV contrast (axial reformatted: left image) (coronal reformatted: right image) demonstrating right-sided pneumothorax (red arrows) and right-sided apical cavitary lesion (yellow arrow). CT: computed tomography; IV: intravenous

The patient returned to the emergency department at our institution with shortness of breath less than a week after being discharged. At this time, he had an oxygen saturation of 92% at room air, which improved to 97% with a 3 L nasal cannula. A chest radiograph showed evidence of a new right-sided spontaneous pneumothorax (Figure 3), which was also demonstrated on CT chest with IV contrast (Figure 4).

**Figure 5:**
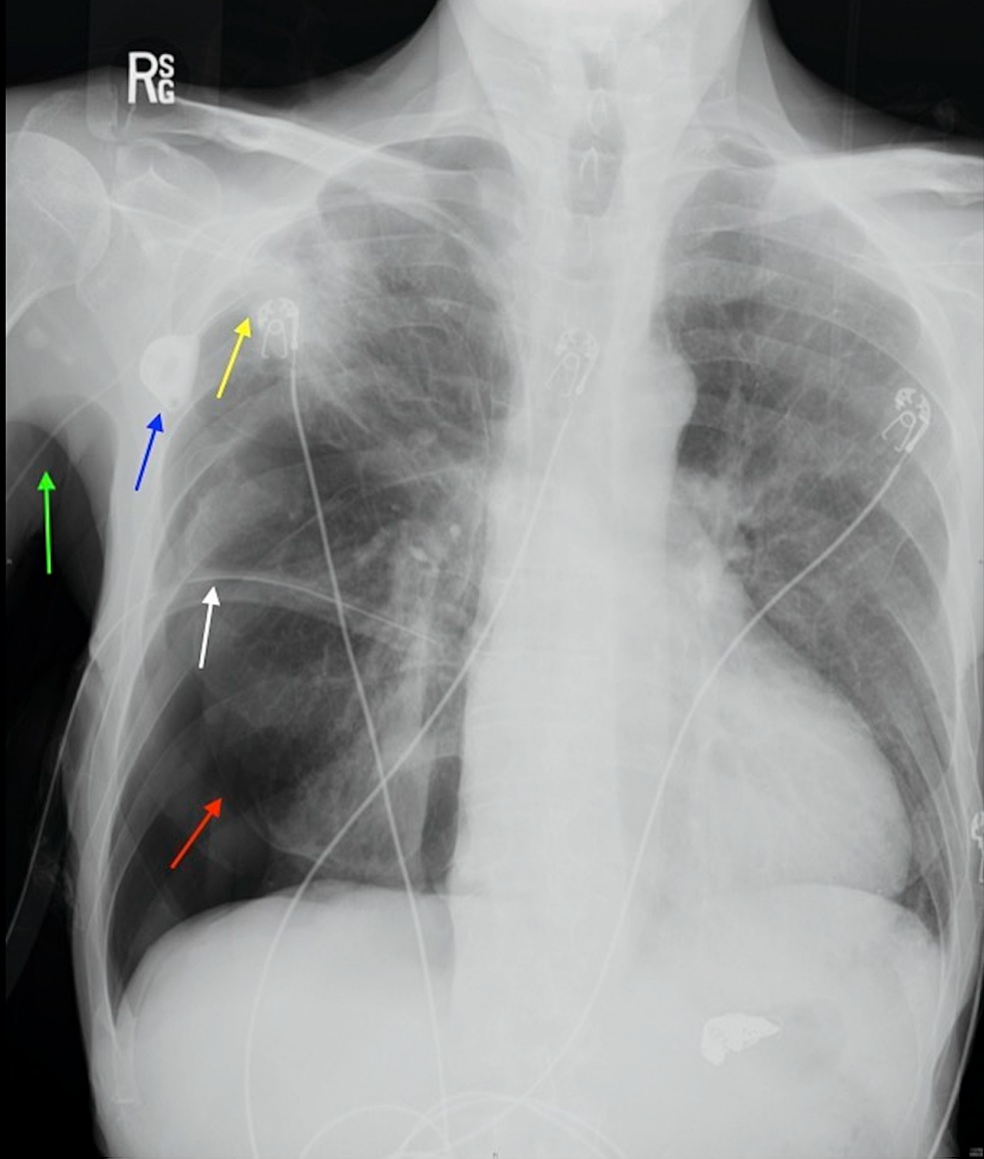
Chest radiograph demonstrating a right-sided pneumothorax (red arrow), a right-sided apical cavitary lesion (yellow arrow), a chemotherapy port (blue arrow), a PICC line (green arrow), and a chest tube (white arrow). PICC: peripherally inserted central catheter

A right-sided chest tube was placed for the treatment of the right-sided pneumothorax (Figure 5).

**Figure 6:**
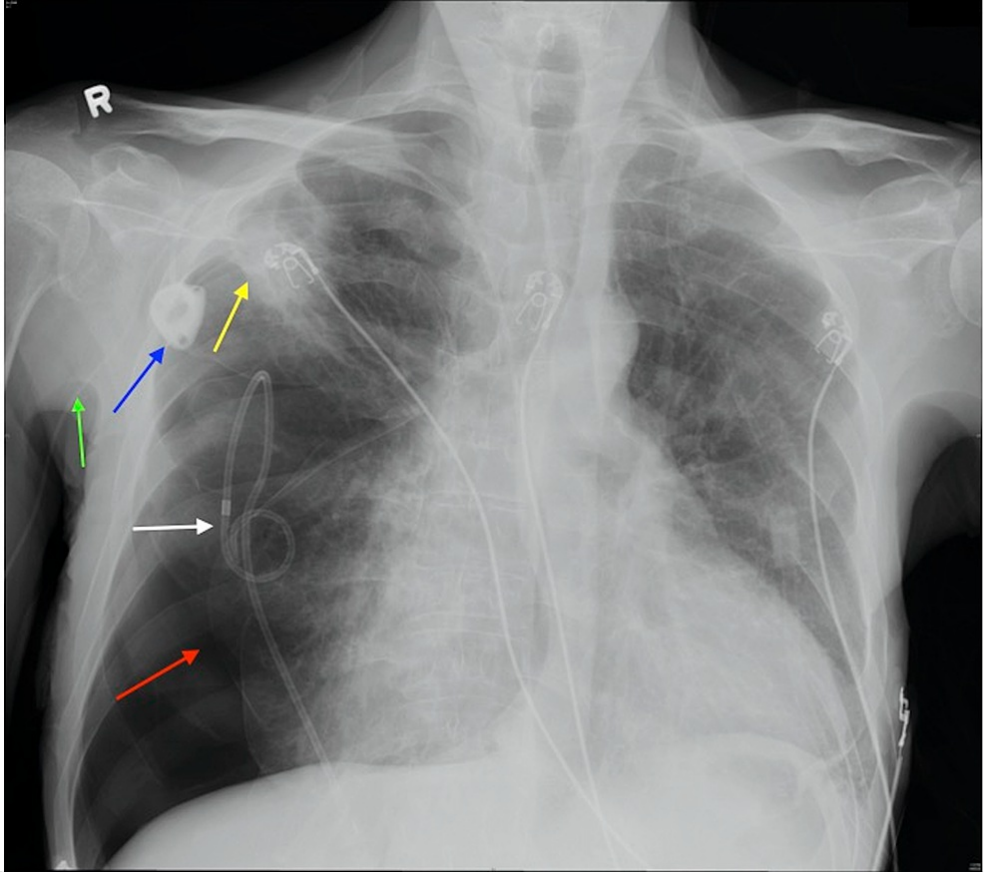
Chest radiograph demonstrating a right-sided pneumothorax (red arrow), a right-sided apical cavitary lesion (yellow arrow), a chemotherapy port (blue arrow), a PICC line (green arrow), and a pigtail catheter (white arrow). PICC: peripherally inserted central catheter

Daily chest radiographs were obtained to monitor the status of the pneumothorax. It continued to enlarge secondary to subsequent malpositioning of the chest tube. At this time, interventional radiology was consulted. A pigtail catheter was placed and the malpositioned chest tube was removed (Figure 6).

Following pigtail catheter placement, the pneumothorax resolved within a week, and the catheter was removed without complications. A repeat chest radiograph demonstrated the resolution of the pneumothorax. The patient’s oxygen saturation improved to 98% on room air.

Amikacin was changed from IV administration to the inhaled formulation to avoid nephrotoxicity on discharge. The patient was additionally sent home with voriconazole, azithromycin, and ethambutol. He was instructed to continue with close follow-up at our Infectious Disease Clinic.

## Discussion

Co-infection in the respiratory tract between *Aspergillus* and *Mycobacteria*, particularly, nontuberculous mycobacteria (NTM) is a rare phenomenon, and its clinical significance has not been adequately defined and needs further research to help guide clinicians make clinical decisions when co-infection arises, as in our patient. Patients with NTM lung infection who also have CPA need to be identified by clinicians as they have worse clinical outcomes than those with NTM lung disease alone [3,4]. The majority of the patients with a mycobacterial infection who also have or later develop CPA typically have *A. fumigatus* or other *Aspergillus *species rather than *A. flavus* like in our patient [5-8]. Although *A. flavus *has not been traditionally mentioned as part of the diagnostic workup for CPA, our patient presenting with CPA secondary to *A. flavus *suggests there may be a benefit in including *A. flavus* serology in the diagnostic workup of CPA, even in areas where it is not typically endemic [9].

## Conclusions

Although rare, CPA has been reported before in patients with NTM lung disease; recognizing this is extremely important for clinicians due to higher mortality in patients with NTM lung disease who are co-infected with CPA. *A. flavus* serology should be included in the diagnostic workup of patients with CPA due to its clinical significance, as demonstrated by our patient having CPA secondary to *A. flavus*.
